# Factors associated with mortality among laboratory-diagnosed drug-resistant tuberculosis patients on treatment, KwaZulu-Natal Province, 2017-2019

**DOI:** 10.11604/pamj.2024.47.181.34571

**Published:** 2024-04-11

**Authors:** Moshibudi Poncho Phafane, Jacqueline Ngozo, Zanele Radebe, Elizabeth Lutge, Joy Ebonwu

**Affiliations:** 1 Division of Public Health Surveillance and Response, National Institute for Communicable Diseases, Johannesburg, South Africa,; 2 Tuberculosis Programme, KwaZulu-Natal Provincial Department of Health, Pietermaritzburg, South Africa,; 3 Epidemiology and Health Research and Knowledge Management, KwaZulu-Natal Provincial Department of Health, Pietermaritzburg, South Africa

**Keywords:** Retrospective studies, tuberculosis, multidrug-resistant, bedaquiline, co-infection, risk factors, protective factors, male, aged, cause of death

## Abstract

**Introduction:**

tuberculosis (TB) remains a leading cause of death in South Africa. KwaZulu-Natal (KZN) is one of the provinces with a high burden of TB/drug-resistant TB cases and deaths. We determined predictors for mortality among drug-resistant TB patients on treatment in KZN province.

**Methods:**

we conducted a retrospective cohort study using secondary data from the Electronic Drug-Resistant Tuberculosis Register. We used a modified Poisson regression model with robust standard errors to determine predictors for drug-resistant TB mortality.

**Results:**

of the 7,692 eligible patients, 1,234 (16.0%) died. Males predominated (707, 57.3%) and the median age was 36 years (Interquartlile Range: 29-45 years). The majority (978, 79.2%) were HIV-TB co-infected with 911 (93%) on antiretroviral treatment (ART). The predictors included HIV-TB co-infection without ART (aIRR 3.4; 95% CI: 2.3-5.1), unknown ART status (aIRR: 1.8; 95% CI: 1.4-2.3), aged ≥60 years (aIRR: 2.1; 95% CI: 1.6-2.7), previous drug-resistant TB (aIRR: 1.5; 95% CI: 1.2-1.8) and exposure to second-line drugs (aIRR: 1.7; 95% CI: 1.4-2.0). Other predictors were hospitalization during treatment initiation (aIRR 2.5; 95% CI 2.0-3.1), initiation in other treatment facilities (aIRR: 2.2; 95% CI: 1.6-2.9) and rifampicin-resistant (aIRR: 1.2; 95% CI: 1.1-1.4). Bedaquiline fumarate was a significant protective factor against death (aIRR: 0.5; 95% CI: 0.4-0.5).

**Conclusion:**

older age, HIV co-infection without ART, hospitalization for treatment initiation, exposure to second-line drugs and a previous episode of drug-resistant TB were predictors for DR-TB mortality. Early treatment initiation and provision of antiretroviral treatment for all co-infected patients may reduce DR-TB mortality in the Province.

## Introduction

Tuberculosis (TB) remains a major cause of morbidity and mortality despite being curable and preventable. According to the World Health Organization (WHO) 2020 global TB report, an estimated 10 million people were diagnosed with TB and mainly (56%) among adult men in 2019. Among all those affected, people living with human immunodeficiency virus (HIV) accounted for 8.2%. Geographically, 25% of the diagnosed TB cases were in Africa. About 1.4 million deaths due to TB, including 208,000 among TB-HIV co-infected people were reported in 2019 [1]. The trend in mortality is a useful indicator for measuring progress towards ending TB [2]. The management of TB is compounded by the complexity of resistance to anti-TB drugs [3,4]. Rifampicin-resistant TB (RR-TB) requires treatment with second-line drugs and includes multidrug-resistant TB (MDR-TB) that is resistant to both rifampicin and isoniazid. The treatment is longer, more toxic and costly, and has poorer outcomes than for drug-sensitive TB [5-9]. The MDR-TB estimate is 3.3% among new TB cases and 18% among previously treated cases and the treatment outcome was hampered by high mortality (15%) and loss to follow-up (16%), leaving a success rate of 57% [10].

South Africa is among eight countries that account for two-thirds of TB infections and 14 countries with the highest burden of MDR-TB and TB/HIV co-morbidity in the world. The WHO statistics give an estimated 360,000 incident TB cases (615 per 100,000 population), with HIV co-infection rate of 58% (209,000 cases) and 14,000 new cases of MDR/RR-TB in 2019 [11]. The first national TB prevalence survey started in 2017, and confirmed the estimated high burden of TB, at a rate of 737 cases per 100,000 population. Tuberculosis remains a leading cause of death in South Africa, with an estimated 58,000 deaths, 62% (36,000) of whom were HIV positive in 2019 [12]. KwaZulu-Natal is a high burden TB/DR-TB province in South Africa, with a reported TB incidence rate of 685 cases per 100,000 population in 2015 [13]. The high prevalence of HIV in the province has impacted greatly on its TB burden [13].

Over the past decade, the South African TB programme has put efforts into improving case finding and management. Of note is the widely implemented GeneXpert MTB/RIF (GXP) assay for early RR-TB diagnosis and prompt linkage to care, which is vital to control the spread of DR-TB. However, a recent study conducted in KwaZulu-Natal showed it had no significant impact on the outcomes of patients with MDR-TB [14]. A similar finding was also observed in a cohort in Ukraine [10]. Documented predictors of mortality in DR-TB patients from studies in South Africa and other parts of the world include HIV co-infection [12,14-16], previous TB treatment [10,14,15,17,18], older age [7,14,19] and hospital admissions [15]. Therefore, understanding the risk factors associated with DR-TB mortality in a high TB/HIV setting, years after concerted efforts have been put in place to reform the TB control programme, is critical for informed decisions. Our study determined predictors for mortality among laboratory-diagnosed drug-resistant tuberculosis patients initiated on treatment in KwaZulu-Natal Province.

## Methods

**Study design and setting:** a retrospective cohort study was conducted using the South African Electronic Drug-Resistant Tuberculosis Register (EDRweb) database. We analysed secondary data collected in public facilities from the 11 districts of KwaZulu-Natal Province. KwaZulu-Natal Province is situated in the southeastern part of South Africa. The north side of the province is bordered by Swaziland and Mozambique, to the east by the Indian Ocean, to the south by Eastern Cape Province, to the west by Lesotho and Free State province, and on the northwest by Mpumalanga province. Within KwaZulu-Natal is an enclave of Eastern Cape Province consisting of the eastern portion of the former Griqualand East (around uMzimkhulu). KwaZulu-Natal is the second largest province in the country by population (11 289 086, mid-year 2020). The province is also one of the provinces with the highest burden of TB (including drug-resistant TB) and HIV (AIDS).

### Study population

***Inclusion criteria:*** the population included all laboratory-confirmed drug-resistant TB patients, aged 18 years and above and registered on the EDRweb from January 2017-December 2019. The patients should be on DR-TB treatment and have outcomes after initiation of treatment.

***Exclusion criteria:*** all the laboratory-confirmed drug-resistant patients on treatment without recorded outcomes and/or still on treatment. The study also excluded patients below 18 years of age.

**Data collection and management:** EDRweb is a web-based software central database that allows authorized users to access and enter data for drug-resistant (DR) TB units. EDRweb is used for the surveillance and management of DR-TB. The database contains key information which is readily displayed to allow for prompt decision-making and to identify gaps and where intervention is required. The data captured covers demographic, clinical, pharmacy (DR-TB treatment) and laboratory. Demographic data includes patient names, district, facility, province, residential address, age and sex. Clinical data includes baseline diagnostic testing conducted, baseline smear and culture results, monthly smear and culture monitoring, HIV and ART status. Patient monitoring and management data include current regimen, regimen changed to, Drug Susceptibility Test (DST) data, as well as sputum monitoring data.

**Data analysis:** the data from EDRweb were exported to an Excel spreadsheet for initial cleaning. We deduplicated and deidentified the data using the name, date of birth and identification number. Data were then exported into STATA® 17.0 [Stata Corporation, College Station, Texas, United States] for further cleaning and analysis. The primary outcome of the study was death after anti-TB treatment initiation. We described the characteristics of patients registered on EDRWEB using summary statistics. Continuous variables were summarised using medians and interquartile range. Frequency tables were used to display categorical data. We used proportions to calculate the frequency of death and related characteristics. A modified Poisson regression model with robust standard errors was used to determine the incidence risk ratios (IRR) and predictors of mortality in patients on drug-resistant TB treatment. The model was used to produce incidence risk ratios (IRR) and the corresponding 95% confidence intervals for the predictors of mortality. We used the variance inflation factor (VIF) to examine multicollinearity between variables. All the variables had VIF values of less than 10. The list of all the predictor variables was drawn first, then the association between each variable and the outcome examined per variable (univariate analysis). The variables that indicated the possible association were selected. A manual forward stepwise method was used to identify the independent predictor variables. We used a p-value of 0.2 as the cut-off value in univariate analysis for inclusion in the multivariate model. In the multivariate analysis, the variables were entered by starting with the one with the smallest p-value. The p-value < 0.05 was considered statistically significant in the multivariate analysis. The univariate and a multivariate modified Poisson regression model was used to analyse the relationship between death and the following variables: demographic variables (age, sex, district, treatment start site) and clinical variables (anti-TB drug history, HIV status, and antiretroviral therapy status).

**Ethical considerations:** the study used ethical clearance from the NICD. The NICD has ethical clearance for essential communicable disease surveillance and outbreak response investigation activities from the University of the Witwatersrand´s Human Research Ethics Committee (Medical) (M160667). The KwaZulu-Natal Provincial Department of Health Research Ethics (KZ 202109-036) also approved the study.

**Funding:** the National Institute for Communicable Diseases and the KwaZulu-Natal Provincial Department of Health funded this study.

## Results

**Figure 1:**
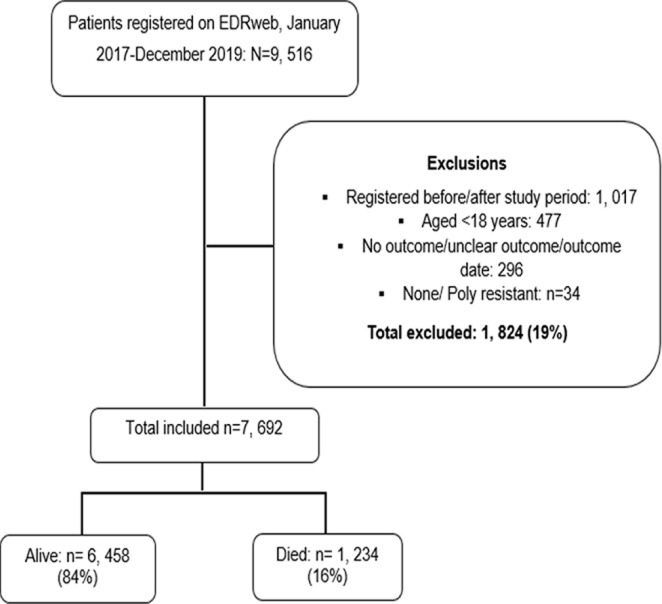
inclusion and exclusion of drug-resistant TB patients, KwaZulu-Natal, January 2017-December 2019

A cumulative total of 9,516 patients were registered on the EDRweb register between 2017 and 2019. We analysed data for 7,692 (81%) patients with laboratory-confirmed drug-resistant TB after excluding patients who did not meet the inclusion criteria (Figure 1).

### Baseline characteristics of DR-TB patients

Of the 7,692 eligible patients, 1,234 (16.0%) had died (Figure 1). The median age at DR-TB treatment initiation was 36 years (interquartile range (IQR): 29-45 years) and 58.3% (4,488) of the patients were male. Although males predominated in all age groups in our study, there were more females (1,084, 53.0%) among patients aged between 18-29 years. Patients diagnosed with rifampicin-resistant TB predominated the study (3,542, 46.0%). Above two-thirds of the patients, 5,852 (76.1%) were HIV-TB co-infected and 5,651 (96.6%) of this subpopulation had been initiated onto ART. The majority of patients had pulmonary TB (7,504, 97.6%) and less than a percentage was unspecified (20). Above half of the patients were registered as newly diagnosed drug-resistant TB patients (4,350). Other patients were treated after relapse (2,109, 27.4%) and lost to follow-up (457, 6.0%). The majority of the patients were on a short regimen (4,483, 58.3%) (Table 1).

**Table 1:** baseline characteristics of DR-TB patients, KwaZulu-Natal Province, 2017-2019

Variable		Total (n=7,692), n (%)
**Age in years**	18-29	2, 030 (26.4)
	30-44	3, 729 (48.5)
	45-59	1,531 (20.0)
	60+	402 (5.2)
**Sex**	Female	3, 204 (41.6)
	Male	4, 488 (58.4)
**Treatment initiation year**	2017	2, 971 (38.6)
	2018	2, 644 (34.4)
	2019	2, 077 (27.0)
**HIV Status**	Negative	1, 805 (23.5)
	Positive	5, 852 (76.1)
	Unknown	35 (0.4)
**HIV-positive on ART**	Yes	5, 651 (96.6)
	No	17 (0.3)
	Unspecified	184 (3.1)
**Drug-resistant type**	Rifampicin Resistant	3, 542 (46.1)
	Laboratory MDR-TB	3, 380 (43.9)
	Pre XDR	480 (6.2)
	XDR-TB	290 (3.8)
**Treatment history**	New patient	4, 353 (56.6)
	Treatment post relapse	2, 109 (27.4)
	Treatment post LTFU	457 (5.9)
	Treatment failure	705 (9.2)
	Other	68 (0.9)
**Previous TB drug history**	New	4, 350 (56.5)
	1st line drugs	2, 797 (36.4)
	2nd line drugs	526 (6.8)
	Unknown	19 (0.2)
**Treatment initiation site**	DR-TB unit	6, 050 (78.6)
	Community level	957 (12.4)
	Other	685 (8.9)
**Type of TB**	Pulmonary	7, 504 (97.6)
	Extra-pulmonary	168 (2.2)
	Unknown	20 (0.3)
**On short regimen**	No	3, 209 (41.7)
	Yes	4, 483 (58.3)
**District**	Amajuba	308 (4.0)
	Ethekwini	3, 415 (44.4)
	Harry Gwala	251 (3.3)
	iLembe	339 (4.4)
	King Cetshwayo	652 (8.5)
	Ugu	566 (7.4)
	Umgungundlovu	666 (8.7)
	Umkhanyakude	484 (6.3)
	Umzinyathi	242 (3.1)
	Uthukela	295 (3.8)
	Zululand	474 (6.2)

### Description of drug-resistant patients by mortality status

The median age for the decedents was 39 years (IQR: 31-50 years) as compared to the survivors (median age: 35 years, IQR: 29-44 years). Above two-thirds of the deaths were patients who initiated treatment in 2017 (522, 42.3%). Among the HIV-TB co-infected subpopulation, the majority of the patients who survived were on ART (4,740, 83.1%). Rifampicin-resistant diagnosed patients contributed to more than half of the deaths (53.0%, n=659). The majority of the decedents were hospitalized during drug-resistant treatment initiation (1,064, 86.2%). There was a significant difference between age groups in those who have died and those who survived (p value<0.001) (Table 2).

**Table 2:** sociodemographic and clinical characteristics of DR-TB patients by mortality status, KwaZulu-Natal Province, 2017-2019

Variable		Total (n=7,692), n (%)	Alive (n=6,458), n (%)	Died (n=1,234), n (%)	p-value
**Age in years**	18-29	2, 030 (26.4)	1, 778 (27.5)	252 (20.4)	<0.001
	30-44	3, 729 (48.5)	3, 183 (49.3)	546 (44.3)	
	45-59	1,531 (20.0)	1, 259 (19.5)	272 (22.0)	
	60+	402 (5.2)	238 (3.7)	164 (13.3)	
**Sex**	Female	3, 204 (41.6)	2, 677 (41.4)	527 (42.7)	0.413
	Male	4, 488 (58.4)	3, 781 (58.6)	707 (57.3)	
**Treatment initiation year**	2017	2, 971 (38.6)	2, 449 (38.0)	522 (42.3)	0.015
	2018	2, 644 (34.4)	2, 244 (34.7)	400 (32.4)	
	2019	2, 077 (27.0)	1, 765 (27.3)	312 (25.3)	
**HIV Status**	Negative	1, 805 (23.5)	1, 554 (24.1)	251 (20.3)	0.017
	Positive	5, 852 (76.1)	4, 874 (75.5)	978 (79.3)	
	Unknown	35 (0.4)	30 (0.4)	5 (0.4)	
**HIV-positive on ART**	Yes	5, 651 (96.6)	4, 740 (97.2)	911 (93.1)	<0.001
	No	17 (0.3)	6 (0.1)	11 (1.1)	
	Unspecified	184 (3.1)	128 (2.6)	56 (5.7)	
**Drug-resistant type**	Rifampicin Resistant	3, 542 (46.1)	2, 883 (44.6)	659 (53.4)	<0.001
	Laboratory MDR-TB	3, 380 (43.9)	2, 916 (45.2)	464 (37.6)	
	Pre XDR	480 (6.2)	414 (6.4)	66 (5.3)	
	XDR-TB	290 (3.8)	245 (3.8)	45 (3.7)	
**Treatment history**	New patient	4, 353 (56.6)	3, 705 957.4)	648 (52.5)	0.001
	Treatment post relapse	2, 109 (27.4)	1, 759 (27.2)	350 (28.4)	
	Treatment post LTFU	457 (5.9)	356 (5.5)	101 (8.2)	
	Treatment failure	705 (9.2)	584 (9.0)	121 (9.8)	
	Other	68 (0.9)	54 (0.8)	14 (1.1)	
**Previous TB drug history**	New	4, 350 (56.5)	3, 704 (57.4)	646 (52.3)	<0.001
	1st line drugs	2, 797 (36.4)	2, 335 (36.2)	462 (37.4)	
	2nd line drugs	526 (6.8)	401 (6.2)	125 (10.1)	
	Unknown	19 (0.2)	18 (0.3)	1 (0.1)	
**Treatment initiation site**	DR-TB unit	6, 050 (78.6)	4, 986 (77.2)	1, 064 (86.2)	<0.001
	Community level	957 (12.4)	892 (13.8)	65 (5.3)	
	Other	685 (8.9)	580 (9.0)	105 (8.5)	
**Type of TB**	Pulmonary	7, 504 (97.6)	6, 295 (97.5)	1, 209 (98.0)	0.569
	Extra-pulmonary	168 (2.2)	146 (2.3)	22 (1.8)	
	Unknown	20 (0.3)	17 (0.3)	3 (0.2)	
**On short regimen**	No	3, 209 (41.7)	2, 689 (41.6)	520 (42.1)	0.744
	Yes	4, 483 (58.3)	3, 769 (58.4)	714 (57.9)	
**District**	Amajuba	308 (4.0)	232 (3.6)	76 (6.2)	<0.001
	Ethekwini	3, 415 (44.4)	3, 036 (47.0)	379 (30.7)	
	Harry Gwala	251 (3.3)	198 (3.1)	53 (4.3)	
	iLembe	339 (4.4)	281 (4.3)	58 (4.7)	
	King Cetshwayo	652 (8.5)	526 (8.1)	126 (10.2)	
	Ugu	566 (7.4)	455 (7.0)	111 (9.0)	
	Umgungundlovu	666 (8.7)	541 (8.4)	125 (10.1)	
	Umkhanyakude	484 (6.3)	404 (6.3)	80 (6.5)	
	Umzinyathi	242 (3.1)	173 (2.7)	69 (5.6)	
	Uthukela	295 (3.8)	230 (3.6)	65 (5.3)	
	Zululand	474 (6.2)	382 (5.9)	92 (7.5)	

### Description of treatment outcomes by drug-resistant type

The median age for RR-TB drug-resistant was 39 years (IQR: 31-49 years), MDR-TB (40 years, IQR: 31-52 years), pre-XDR-TB (35 years, 29-43 years) and XDR-TB (41 years, IQR: 33-50 years) respectively. More than half of the patients were cured (4,157, 54.0%). The proportion of patients who were cured was above 50% in all the drug-resistant types (Table 3). Although RR-TB patients predominated, MDR-TB (1,935, 46.6%) contributed the highest proportion of patients who were cured than those diagnosed with other drug-resistant types. Among patients who had treatment failure, MDR-TB patients contributed the highest proportion (39, 43.1%) followed by RR-TB (24, 24.7%) (Table 3).

**Table 3:** treatment outcomes by drug-resistant type, KwaZulu-Natal, January 2017-December 2019

Final Treatment Outcome	All patients	RR-TB	MDR-TB	Pre XDR-TB	XDR-TB
**Cured (C)**	4, 157 (54.0)	1, 825 (51.5)	1, 935 (57.2)	246 (51.2)	151 (52.1)
**Died (D)**	1, 234 (16.0)	659 (18.6)	464 (13.7)	66 (13.7)	45 (15.5)
**Loss to Follow-Up**	1, 011 (13.1)	480 (13.5)	416 (12.3)	78 (16.2)	37 (12.8)
**Treatment Completed (TC)**	1, 200 (15.6)	554 (15.6)	526 (15.6)	70 (14.6)	50 (17.2)
**Treatment Failure (TF)**	90 (1.2)	24 (0.7)	39 (1.1)	20 (4.2)	7 (2.4)
**Total outcomes**	n=7, 692	n=3, 542	n=3, 380	n=480	n=290

### Description of DR-TB deaths

A cumulative 1,234 (16.0%) patients diagnosed with DR-TB died. Males predominated in the proportion of deaths among different age groups except in decedents between 18-29 years of age (150, 59%). Of the total deaths, 978 (79.2%) were HIV-TB co-infected. Among HIV-TB co-infected patients who died, 911 (93.1%) were on ART. Of the decedents who were not initiated on ART, the majority were males (16, 72%). A total of 646 (52.3%) decedents were registered as newly diagnosed drug-resistant TB patients (Table 3). Over half of the patients were on short regimens (714, 58.1%). Slightly above half of the deaths (659, 53%) were diagnosed with RR-TB (Table 3). On further analysis, deaths were stratified by drug-resistant type. Male patients contributed to two-thirds of the XDR-TB related deaths (30, 66.7%). Patients aged between 30-44 years contributed more deaths in all drug-resistant TB types than other age groups. Above 70% of the deaths in all resistant types were HIV-TB co-infected and above 95% on ART4.

### Factors associated with mortality among DR-TB patients

Using Fisher´s exact to test for statistical significance in the frequency of mortality, there was a significant difference in the frequency of deaths among age groups, those who had previous drug history, drug-resistant type, treatment initiation site and treatment initiation districts (p-value: < 0.001). We observed a significant difference in the frequency of mortality among those who initiated treatment in 2017, 2018 and 2019. There was no remarkable difference in frequency of deaths in relation to sex, having pulmonary or extra-pulmonary TB and either patient was on a short regimen or not (Table 1). On multivariate analysis, HIV-TB co-infection without ART (aIRR 3.4; 95% CI: 2.3-5.1; p-value < 0.001) and those with unknown ART statuses (aIRR: 1.8; 95% CI: 1.4-2.3; p-value < 0.001) had significant association with mortality during DR-TB treatment. Patients aged 60 years and above were at higher risk of mortality (aIRR: 2.1; 95% CI: 1.6-2.7; p-value < 0.001). The patients who had a previous episode of drug-resistant TB (aIRR: 1.5; 95% CI: 1.2-1.8; p-value: 0.001) and exposure to second-line drugs (aIRR: 1.7; 95% CI: 1.4-2.0; p-value: < 0.001) also had significant risk of mortality. Other predictors included hospitalization during DR-TB treatment initiation (aIRR 2.5; 95% CI 2.0-3.1; p-value: < 0.001), treatment initiation in other drug-resistant initiation facilities (aIRR: 2.2; 95% CI: 1.6-2.9; p-value: < 0.001) and diagnosis with RR-TB (aIRR: 1.2; 95% CI: 1.1-1.4; p-value: < 0.001). Treatment with bedaquiline fumarate was a significant protective factor against death amongst patients on TB-drug resistant treatment (aIRR: 0.5; 95% CI: 0.4-0.5; p-value: <0.001) (Table 4).

**Table 4:** predictors of mortality among DR-TB patients, KwaZulu-Natal, 2017-2019

Variable		Univariate analysis		Multivariate analysis	
		IRR (95% CI)	P value	aIRR (95% CI)	p-value
**Age in years**	15-29	Ref*		Ref*	
	30-44	1.2 (1.0-1.3))	0.020	1.0 (0.9-1.2)	0.967
	45-59	1.4 (1.2-1.7)	**<0.001**	1.2 (1.0-1.5)	0.010
	60+	3.3 (2.8-3.8)	**<0.001**	2.1 (1.6-2.7)	**<0.001**
**Sex**	Female	Ref*			
	Male	1.0 (0.9-1.1)	0.413		
**Registration year**	2017	Ref*			
	2018	0.9 (0.8-1.0)	**0.014**		
	2019	0.8 (0.8-1.0)	**0.017**		
**HIV Status**	Negative	Ref*			
	Positive	1.2 (1.2-1.4)	**0.005**		
	Unknown status	1.0 (0.2-2.1)	0.949		
**On ART**	Yes	Ref*			
	No	4.0 (2.8-5.7)	**<0.001**	3.4 (2.3-5.1)	**<0.001**
	Unknown	1.9 (1.5-2.4)	**<0.001**	1.8 (1.4-2.3)	**<0.001**
**Drug-resistant type**	MDR-TB	Ref*			
	Rifampicin Resistant	1.3 1.1-1.7)	**0.011**	1.2 (1.1-1.4)	**<0.001**
	Pre XDR	1.0 (0.8-1.3)	0.989	1.1 (0.8-1.4)	0.652
	XDR-TB	1.1 (0.8-1.6)	0.498	1.1 (0.8-1.5)	0.620
**Treatment initiation site**	Community-level	Ref*			
	DR-TB Hospital	2.6 (2.0-3.3)	**<0.001**	2.5 (2.0-3.2)	**<0.001**
	Other healh care sites	2.2 (1.7-3.0)	**<0.001**	2.0 (1.4-2.7)	**<0.001**
**On BDQ**	No	Ref*			
	Yes	0.5 (0.9-1.1)	**<0.001**	0.5 (0.4-0.5)	**<0.001**
**Previous drug history**	Newly diagnosed	Ref*			
	1st line drugs	1.1 (1.0-1.2)	0.057	1.1 (1.0-1.2)	0.040
	2nd line drugs	1.6 (1.3-1.9)	**<0.001**	1.7 (1.4-2.0)	**<0.001**
	Unspecified	0.3 (0.1-2.4)	0.287	0.3 (0.1-2.0)	0.214
**Previous drug-resistant TB**	No	Ref*			
	Yes	1.6 (1.4-1.9)	**<0.001**	1.5 (1.2-1.8)	**0.001**
**On short regimen**	No	Ref*			
	Yes	(1.0-1.1)	0.744		
**Treatment history**	New patient	Ref*			
	Treatment post relapse	1.1 (1.0-1.2)	0.074		
	Treatment post LTFU	1.5 (1.2-1.8)	**<0.001**		
	Treatment failure	1.1 (1.0-1.4)	0.115		
	Other	1.4 (0.8-2.2)	0.178		

## Discussion

In this study, we determined predictors for mortality among DR-TB patients in KwaZulu-Natal Province over three years. We defined a DR-TB patient (using the WHO definition) as any TB case caused by *Mycobacterium tuberculosis* resistant to at least one anti-TB drug. The predictors determined by this study included older age, TB-HIV co-infection without ART and those with undocumented ART statuses, previous history of drug-resistant TB, history of second-line anti-TB treatment, diagnosis with RR-TB and being hospitalized in a DR-TB hospital setting during treatment initiation. Patients aged 60 years and above contributed the lowest proportion of DR-TB patients and deaths; however, we found that these patients were at higher risk for mortality. This finding is consistent with the two studies conducted in different settings in South Africa [15,18]. The studies determined the age of 60 years and older as a significant predictor of mortality in drug-resistant TB patients. An increase in age is associated with a weakened immune system and the increased likelihood of having other developing underlying conditions [19-21]. The systematic review conducted in Ethiopia indicated that as the age increases in one unit, the incidence of mortality increases by a percentage [21]. In our study, we however only assessed the HIV co-infection as comorbidity on DR-TB mortality.

Because our study was conducted post-universal ART coverage era, HIV-TB co-infection was not associated with mortality among DR-TB patients. However, being HIV-infected without ART or with unknown ART status were significantly associated with mortality among drug-resistant TB patients. The study also found that males contributed the majority of the decedents who were not initiated on ART. However, there was no association between mortality and being male or female determined in this study. The findings on the association of mortality and HIV-TB co-infection without ART and/or undocumented ART status were consistent with other studies [15,18,22-24]. The study conducted in Zimbabwe indicated that mortality was most common in the intensive phase of anti-TB treatment in those who were HIV-positive without documented ART initiation. The study also determined that ART had a protective effect against mortality among co-infected patients on drug-resistant treatment [23]. In contrast, the study conducted in a rural area in KwaZulu-Natal determined that ART was effective in MDR-TB patients, but not in XDR-TB patients [25]. In our study, ART was protective in all drug-resistant types.

Our study also determined that previous history of drug-resistant TB and a history of treatment with second-line anti-TB drugs increased the likelihood of mortality compared to newly diagnosed patients. This was consistent with other studies that found mortality notably higher among patients with recurrent TB episodes [23,26-28]. The meta-analysis conducted in Ethiopia, also, found that the history of TB infection and history of treatment with second-line anti-TB drugs increased the risk of death amongst DR-TB patients [21]. Another study conducted in Peru indicated that receipt of fewer prior regimens reduced the rate of death by almost half (Aggressive regimens). The study also found that patients initiated on an aggressive regimen were less likely to die (crude hazard ratio [HR]: 0.62; 95% CI: 0.44, 0.89), compared to those who were on less aggressive regimens. In our study, treatment with bedaquiline fumarate was a significant protective factor against death amongst DR-TB patients. The use of bedaquiline fumarate in MDR-TB treatment is associated with substantial mortality benefits [29]. A key finding in the trial study conducted by Pym *et al*. was that overall mortality declined from 12% to 6.9%, with the use of bedaquiline in treating MDR-TB and XDR-TB patients. Another study (systematic review) conducted by Hatami *et al*. indicated that the use of bedaquiline at treatment initiation and as part of an all-oral regimen may preserve good overall treatment outcomes. The study further indicated that bedaquiline may also improve time to culture conversion and minimize adverse effects, such as hearing loss, associated with the injectable agents [30,31].

Our study also found treatment initiation site as a risk factor for mortality among DR-TB patients. Patients who were initiated on treatment as inpatients had an increased risk of death as compared to those who were initiated onto TB treatment as outpatients. This might be due to the poor prognoses at the start of treatment in those who required admission than those with more favourable prognoses. The study conducted in Canada indicated that delay in diagnosing TB patients may result in patients presenting with a more severe disease at the time of diagnosis [32]. Another study determined being initiated on treatment at a hospital (aIRR = 3.73, 95%CI: 2.23, 6.25) as one of the predictors of unsuccessful outcomes [33]. However, these could be consistent with the South African National Guidelines that prioritize hospitalization for severely ill MDR-TB and XDR-TB patients [34]. Our study had some limitations; therefore, the results should be interpreted with precaution. The data analysed was routinely collected for surveillance purposes. This contributed to an inability to assess the impact of other comorbidities on mortality, the time to death and the proportion of patients initiated in the community that were subsequently admitted into DR-TB hospitals after treatment initiation. Secondly, the incompleteness of data on outcomes for those who were on treatment within the study period might have underestimated the number of deaths. Lastly, the exclusion of those with mono-resistant, poly-resistant and those not confirmed as DR-TB patients may have introduced bias.

## Conclusion

We investigated several predictors of mortality among drug-resistant TB patients in KwaZulu-Natal Province. Though no new findings were determined, this study provides an understanding of the epidemiology as well as risk factors for drug-resistant mortality in the KwaZulu-Natal Province. The study found that older patients (60 years and above), being HIV co-infected without ART, being hospitalised for DR-TB treatment initiation, previous history of DR-TB treatment and having had second-line treatment for TB previously were identified as significant risk factors for DR-TB mortality. Anti-TB drugs should be continuously monitored, in order to document and quantify their effects on TB patients. Integration of TB/HIV services especially on HIV testing and provision of ART for all co-infected patients may contribute to improving the clinical state of TB patients and reduction of mortalities in the KwaZulu-Natal Province. We recommend further studies with more robust data collection and analysis in order to identify incidence and risk factors associated with mortality in patients on anti-TB treatment.

### What is known about this topic

- *Older patients (60 years and above), being HIV co-infected without ART, being hospitalised for DR-TB treatment initiation as significant predictors for mortality in drug-resistant patients;*
- *Male patients predominating the number of deaths in drug-resistant TB patients;*
- *The previous history of drug-resistant TB and history of treatment with second-line anti-TB drugs increased the likelihood of mortality compared to newly diagnosed patients*.

### What this study adds

- *Factors associated with mortality among drug-resistant TB patients on treatment in a TB and HIV highly burdened province in South Africa;*
- *Bedaquiline is a protective factor for mortality in all the drug-resistant TB types;*
- *Variation in treatment initiation site as a significant predictor for drug-resistant TB*.
